# Are torso asymmetry and torso displacements in a computer brace model associated with initial in-brace correction in adolescent idiopathic scoliosis?

**DOI:** 10.1186/s12891-023-06440-8

**Published:** 2023-05-08

**Authors:** Charles M. M. Peeters, Peter A. J. Pijpker, Frits-Hein Wapstra, Diederik H. R. Kempen, Chris Faber

**Affiliations:** 1 Department of Orthopaedics, University of Groningen, University Medical Center Groningen, Hanzeplein 1, 9713 GZ Groningen, The Netherlands; 2 Department of Orthopaedics, & 3D Lab, University of Groningen, University Medical Center Groningen, Groningen, The Netherlands; 3 Department of Orthopaedics, OLVG, Amsterdam, Amsterdam, The Netherlands

**Keywords:** Scoliosis, Brace, In-brace correction, CAD technology, Predictive factors

## Abstract

**Background:**

Lack of initial in-brace correction is strongly predictive for brace treatment failure in adolescent idiopathic scoliosis (AIS) patients. Computer-aided design (CAD) technology could be useful in quantifying the trunk in 3D and brace characteristics in order to further investigate the effect of brace modifications on initial in-brace correction and subsequently long-term brace treatment success. The purpose of this pilot study was to identify parameters obtained from 3D surface scans which influence the initial in-brace correction (IBC) in a Boston brace in patients with AIS.

**Methods:**

Twenty-five AIS patients receiving a CAD-based Boston brace were included in this pilot study consisting of 11 patients with Lenke classification type 1 and 14 with type 5 curves. The degree of torso asymmetry and segmental peak positive and negative torso displacements were analyzed with the use of patients’ 3D surface scans and brace models for potential correlations with IBC.

**Results:**

The mean IBC of the major curve on AP view was 15.9% (SD = 9.1%) for the Lenke type 1 curves, and 20.1% (SD = 13.9%) for the type 5 curves. The degree of torso asymmetry was weakly correlated with patient’s pre-brace major curve Cobb angle and negligible correlated with major curve IBC. Mostly weak or negligible correlations were observed between IBC and the twelve segmental peak displacements for both Lenke type 1 and 5 curves.

**Conclusion:**

Based on the results of this pilot study, the degree of torso asymmetry and segmental peak torso displacements in the brace model alone are not clearly associated with IBC.

**Supplementary Information:**

The online version contains supplementary material available at 10.1186/s12891-023-06440-8.

## Background

Bracing of adolescent idiopathic scoliosis (AIS) is effective to stop progression of the curve in 72% of the patients [1]. The Boston brace is a widely used brace system, which consists of a prefabricated symmetric module that is customized to fit an individual patient’s body shape and spinal curvature [2, 3]. Unfortunately, brace treatment is not successful in every AIS patient. Apart from brace compliance, strong evidence has been reported for lack of initial in-brace correction as a predictive factor for brace treatment failure [4].

Curve type and curve flexibility are the best proven factors influencing this initial in-brace correction, but these patient factors cannot be influenced by the orthotist [5]. Translations generated by the brace on the thorax generally are statistically and linearly related to corresponding corrections of the spine, and a positive correlation has been reported between the correction of the lumbar scoliosis and correction of the lumbar lordosis [6, 7]. To influence these translations generated by the brace, computer-aided design and manufacturing systems (CAD/CAM) combined with or without finite element models (FEM) simulation have been applied. So far, theydo not significantly improve initial in-brace correction compared to a conventional plaster-cast method [5, 8–11]. However, these CAD technologies could be useful in quantifying the trunk in 3D and brace characteristics in order to further investigate the effect of brace modifications on initial in-brace correction and subsequently long-term brace treatment success. The purpose of this pilot study was to identify parameters obtained from 3D surface scans which influence the initial in-brace correction (IBC) in a Boston brace in patients with AIS. The degree of torso asymmetry (i) and segmental peak positive and negative torso displacements (ii) will be analyzed with the use of patients’ 3D surface scans and brace models for potential correlations with IBC.

## Methods

### Patients

This retrospective pilot study was approved by the Medical Ethical Review Board (RR-number: 201800846). Inclusion criteria were: AIS patients aged between 10 and 17 years (i), with a pre-brace Lenke classification type 1 or 5 curve (ii), and a pre-brace Cobb angle of the major curve of 20 degrees or more (iii), undergoing Boston brace treatment manufactured with CAD (iv) [12]. Patients with non-idiopathic scoliosis or previous spine operations were excluded. All eligible patients, retrieved from a database of Boston brace users, were approached for study participation by mail, telephone or at the outpatient clinics. The first 25 patients who gave their informed consent were included in this pilot study.

### Method of measurements

Pre-brace and in-brace standing biplanar low-dose radiographs of the spine were made using EOS®imaging, Paris, France [13, 14]. Two independent observers (CP and CF) determined the Lenke classification of the scoliosis deformities and separately measured the major curve Cobb angle on the anteroposterior (AP) and lateral view [12]. When the difference in Cobb angle between the observers was exceeding 5 degrees, a consensus meeting was planned. In the results, the data are presented as the mean of both observers.

The brace manufacturing process consisted of 3D torso scans from which a virtual brace model was designed. These brace 3D models have been prepared by the orthotist at the time of brace manufacturing. This process included virtual reshaping of the torso scan towards the desired torso, which was then milled out of a foam block, forming a mold for the final brace. For this study all 3D surface scans and brace models of included patients were obtained from the orthotist and analysed by a technical physician from our point-of-care 3D lab (PP), who were both blinded for initial in-brace correction.

**Fig. 1:**
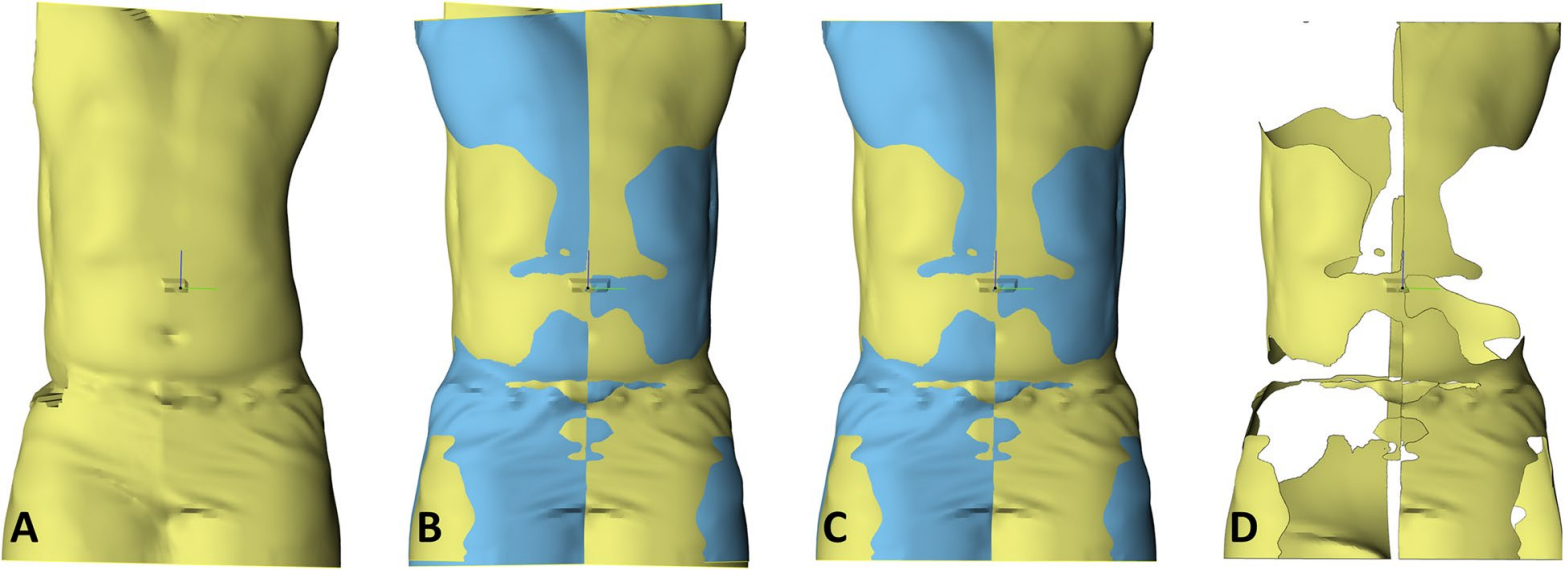
Symmetry analysis showing, **A**) the torso scan in yellow, **B**) registered mirrored torso scan in blue, **C**) the trimmed to equal length, and **D**) the volume between both surfaces

First the asymmetry index was determined for all the torso and brace models. Due to the lack of available standardized methods to assess the torso asymmetry, this study’s method was based on a variety of methods for assessing facial asymmetry [15]. The surface models were imported into 3-matic v12 (Materialise, Belgium, Leuven) (Fig. 1A). First, manual positioning of mirroring planes was performed, the models were then mirrored across these planes. Next, the mirrored models were registered to the original models using the in-software iterative closest point (ICP) algorithm (Fig. 1B). The top and bottom of these models were then trimmed in order to obtain equal length (Fig. 1C). Finally, the volume enclosed between the mirrored model and the original model was measured and divided by the total volume of the original model, providing us with the asymmetry percentage (Fig. 1D). The asymmetry percentage was calculated for the torso as well as the brace models.

**Fig. 2:**
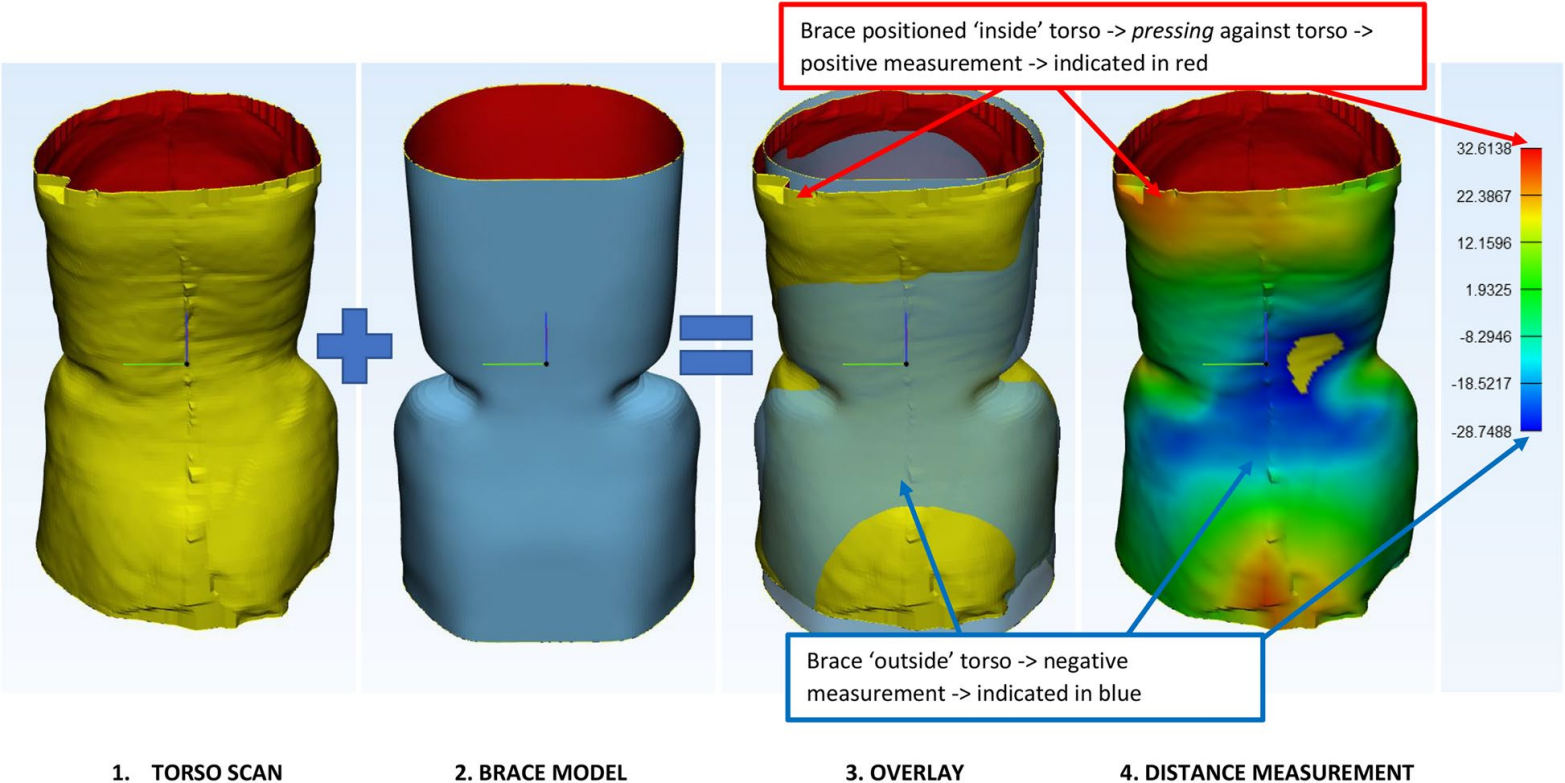
Surface-to-surface measurements. The torso scans and brace model are properly aligned by the orthotist. From each point on the torso scan the distance towards the brace model is measured using an algorithm. At the surface areas where the brace provides space to the torso this results in negative values and a blue color (expansion zone). For the red area there is an opposite effect; here the brace is *pressing* against the torso (pressure zone)

**Fig. 3:**
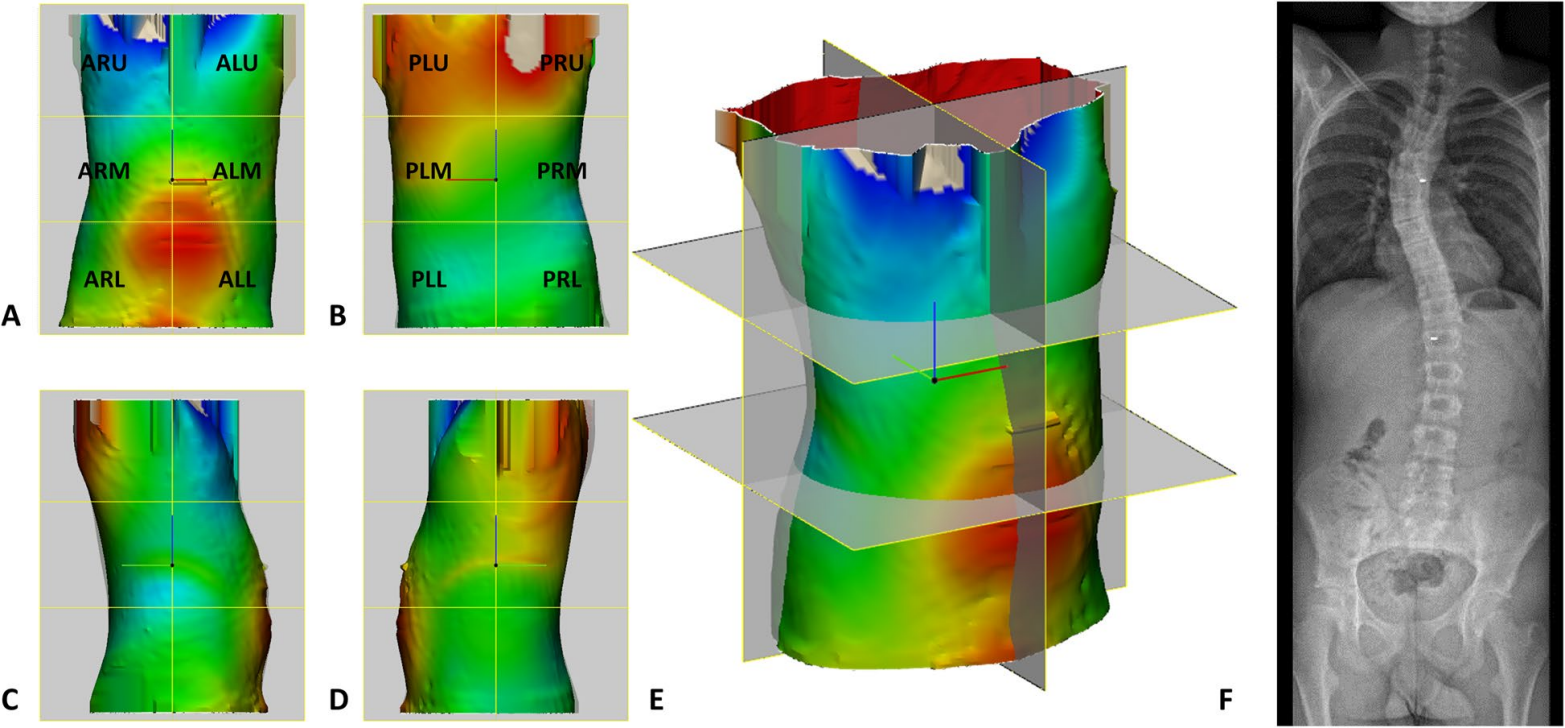
Surface-to-surface measurements (A-E) and EOS radiograph (F) of a typical case, in **A**) anterior, **B**) posterior, **C**) right, **D**) left, **E**) perspective view. Initial in-brace correction = 9.6%. Abbreviations: ALU anterior left upper segment ARU anterior right upper segment, ALM, anterior left midsegment, ARM, anterior right midsegment, ALL, anterior left lower segment, ARL, anterior right lower segment, PLU, posterior left upper segment, PRU, posterior right upper segment, PLM, posterior left midsegment, PRM, posterior right midsegment, PLL, posterior left lower segment, PRL, posterior right lower segment.

The second parameter was based on surface-to-surface distance measurements between the torso scan and the brace model. The surface-to-surface analysis in 3-matic was used to measure the closest distance of each surface point on the torso surface to the nearest neighbouring point on the brace model. At the areas where the brace model was situated ‘inside’ the torso this resulted in a positive value (or red color), and at places where the brace was situated ‘outside’ the torso the algorithm provided with a negative value or a blue color (Fig. 2). I.e. a positive value corresponds to areas where the brace is *pressed* against the torso (pressure zone), and a negative value corresponds with areas where the torso could move away from the brace (expansion zone). For the final analysis the analysis model is divided into 12 segments. Two cross sectional planes are created by 2 planes in the z-direction, creating an upper, middle and lower segment, which are equally divided. A coronal midplane and a sagittal midline then divide the torso into 12 segments (Fig. 3).

### Statistical analyses

Spearman’s rho correlation coefficients were calculated to determine the correlation between.the torso asymmetry index and pre-brace major curve Cobb angle and initial in-brace correction (i), and correlations between segmental peak positive and negative displacements and initial in-brace correction separately for Lenke 1 and 5 curves.A Spearman’s rho of 0.90–1.00 indicates a very strong correlation, a Spearman’s rho of 0.70–0.89 indicates a strong correlation, 0.50–0.69 moderate, 0.26–0.49 weak, and ≤ 0.25 represents little if any correlation [16–18]. IBM SPSS Statistics for Windows, version 23.0 (IBM Corp., Armonk, NY, USA) was used for all statistical analyses.

## Results

### Patient inclusion and characteristics

Twenty-five patients with a mean age of 14 years (SD = 1.5) at start Boston brace treatment were included in this pilot study (Table 1). Eleven patients had a type 1 curve and 14 patients a type 5 curve according to the Lenke classification. All type 1 curves were thoracic right-convex and all type 5 curves were lumbar left-convex. Sixteen patients (64%) were female. The mean pre-brace Cobb angle of the major curve were 38.4 degrees (SD = 14.8) and 30.5 degrees (SD = 5.8) for the type 1 and type 5 curve, respectively. The mean initial in-brace correction of the major curve was 15.9% (SD = 9.1%) for the type 1 curves, and 20.1% (SD = 13.9%) for the type 5 curves. All initial in-brace corrections were the result of the CAD correction without additional padding. If necessary, further improved with adjustment of the brace pads were done by the orthotist after the first in-brace radiograph. These additional corrections by pads were not included in the measurements. The mean time interval between pre-brace and in-brace radiographic follow-up images was 3.3 months (SD = 1.5). Six patients (26%) of which 5 patients (80%) with a Lenke type 1 curve had brace treatment failure, which was defined as indication for surgery.

**Table 1:** Patient characteristics

**Criterion**		**Study population**
Gender, female^a^		16 (64%)
Age at start Boston brace treatment		14.0 ± 1.5
Brace initiation before menarche^a^		6 (38%)
Pre-brace Lenke classification^a ^[12]		
Lenke 1		11 (44%)
Lenke 5		14 (56%)
Pre-brace Nash Moe classification at major curve apex^a ^[19]		
Nash moe 0		1 (4%)
Nash moe 1		6 (24%)
Nash moe 2–3		18 (72%)
Brace treatment failure^a,c^		6 (26%)
	**Lenke 1 (***n* = 11)^b^	**Lenke 5 (***n* = 14)^b^
Pre-bracemajor curve CA on AP view	38.4 ± 14.8	30.5 ± 5.8
Initial in-brace correction major curve on AP view in CA degrees	6.2 ± 3.7	6.0 ± 3.7
Initial in-brace correction major curve on AP view in %	15.9 ± 9.1	20.1 ± 13.9
Pre-brace major curve CA on lateral view	21.2 ± 15.0	48.3 ± 11.9
Initial in-brace correction major curve on lateral view in CA degrees	4.5 ± 5.8	7.0 ± 6.9
Initial in-brace correction major curve on lateral view in %	5.1 ± 81.9	13.7 ± 14.4

*Abbreviations: AP* Anteroposterior, *CA* Cobb angle, n Number of patients with Lenke 1 or Lenke 5 curve, *SD* Standard deviation

^a^Values are presented as number (percentage)

^b^Values are presented as mean ± standard deviation

^c^Brace treatment failure was defined as indication for surgery

### Torso asymmetry, pre-brace Cobb angle and in-brace correction

The mean torso asymmetry index was 5.6% (SD = 1.6) for patients with type 1 curves, and 3.9% (SD = 1.3) for type 5 curves (Table 2). A weak positive correlation was observed between patients’ torso asymmetry index and pre-brace major curve CA on AP view for both type 1 and 5 curves (Spearman’s rho = 0.29 and 0.33, respectively). Little or negligible negative correlation was found between patient’s torso asymmetry index and initial in-brace correction on AP view (Spearman’s rho = -0.08 for Lenke type 1 curves, and Spearman’s rho = -0.14 for type 5 curves, see Table 2).

**Table 2:** Torso asymmetry

**Criterion**	**Lenke 1 (*n* = 11) Mean ± SD**	**Lenke 5 (*n* = 14) Mean ± SD**
Torso asymmetry index in %	5.64 ± 1.60	3.93 ± 1.30
Brace asymmetry index in %	0.18 ± 0.36	0.08 ± 0.11
**Correlation with torso asymmetry index**	**Spearman’s rho**	**Spearman’s rho**
Pre-brace major curve CA on AP view	0.29	0.33
Initial in-brace correction major curve on AP view	-0.08	-0.14
Pre-brace major curve CA on lateral view	-0.29	-0.17
Initial in-brace correction major curve on lateral view	-0.37	-0.06

*Abbreviations: AP* Anteroposterior, *CA* Cobb angle, *n* Number of patients with Lenke 1 or Lenke 5 curve, *SD* Standard deviation

### Peak torso displacement and in-brace correction

For the type 1 curves a strong negative correlation was observed between the peak negative torso displacement in the anterior right midsegment (ARM) and major curve IBC (Spearman’s rho = -0.72, see Table 3). Also, a moderate correlation was observed between the peak positive displacement in the posterior left midsegment (PLM) and IBC (Spearman’s rho = 0.64), and a moderate negative correlation was observed between the peak positive displacement in the anterior right upper segment (ARU) and IBC (Spearman’s rho = -0.51), and the peak negative displacement in the posterior right midsegment (PRM) and IBC (Spearman’s rho = -0.55). Weak or little if any correlation was observed between the other segmental peak positive and negative displacements and IBC (Spearman’s rho < 0.50, see Table 3).

**Table 3:** Correlations between segmental peak positive and negative torso displacements and initial in-brace correction in Lenke 1 curves

Correlation with IBC	Little if any correlation rho ≤ 0.25	Weak correlation rho = 0.26–0.49	Moderate correlation rho = 0.50–0.69	Strong correlation rho = 0.70–0.89
**Peak positive torso displacement**				
ALU		-0.43		
ARU			-0.51	
ALM		0.48		
ARM	0.16			
ALL	-0.21			
ARL		-0.26		
PLU		0.47		
PRU		0.33		
PLM			0.64	
PRM		-0.27		
PLL	0.08			
PRL		0.32		
**Peak negative torso displacement**				
ALU		-0.32		
ARU	-0.10			
ALM		0.28		
ARM				-0.72
ALL	-0.07			
ARL	0.13			
PLU	0.09			
PRU	-0.05			
PLM	-0.13			
PRM			-0.55	
PLL	0.09			
PRL	-0.01			

*Abbreviations*: *IBC* Initial in-brace correction, *rho* Spearman’s rho, *ALU* Anterior left upper segment, *ARU* Anterior right upper segment, *ALM* Anterior left midsegment, *ARM* Anterior right midsegment, *ALL* Anterior left lower segment, *ARL* Anterior right lower segment, *PLU* Posterior left upper segment, *PRU* Posterior right upper segment, *PLM* Posterior left midsegment, *PRM* Posterior right midsegment, *PLL* Posterior left lower segment, *PRL* Posterior right lower segment

For type 5 curves, only weak or negligible correlations were found between the peak positive displacements in the twelve segments and IBC (Table 4). Regarding the peak negative displacements, a strong negative correlation was observed between this displacement in the PLM segment and IBC (Spearman’s rho = -0.85). Also a moderate negative correlation was observed between the peak negative displacement in the posterior left upper segment (PLU) and IBC (Spearman’s rho = -0.54, see Table 4).

**Table 4:** Correlations between segmental peak positive and negative torso displacements and initial in-brace correction in Lenke 5 curves

Correlation with IBC	Little if any correlation rho ≤ 0.25	Weak correlation rho = 0.26–0.49	Moderate correlation rho = 0.50–0.69	Strong correlation rho = 0.70–0.89
**Peak positive torso displacement**				
ALU	-0.01			
ARU	-0.04			
ALM	0.04			
ARM		0.26		
ALL	-0.05			
ARL	-0.24			
PLU	-0.13			
PRU		-0.31		
PLM		-0.34		
PRM	-0.11			
PLL	0.07			
PRL	0.21			
**Peak negative torso displacement**				
ALU	-0.11			
ARU	0.01			
ALM		0.26		
ARM	0.16			
ALL		-0.27		
ARL		-0.49		
PLU			-0.54	
PRU	0.04			
PLM				-0.85
PRM	-0.23			
PLL		-0.37		
PRL	-0.25			

*Abbreviations:* *IBC* Initial in-brace correction, *rho* Spearman’s rho, *ALU* Anterior left upper segment, *ARU* Anterior right upper segment, *ALM* Anterior left midsegment, *ARM* Anterior right midsegment, *ALL* Anterior left lower segment, *ARL* Anterior right lower segment, *PLU* Posterior left upper segment, *PRU* Posterior right upper segment, *PLM* Posterior left midsegment, *PRM* posterior right midsegment, *PLL* Posterior left lower segment, *PRL* Posterior right lower segment

Correlations between segmental peak positive and negative displacement and IBC on lateral radiographs for both type 1 and 5 curves are presented in the supplementary data Tables 1 and 2. Besides a moderate negative correlation between the peak positive displacement in the anterior left lower segment (ALL) and major curve IBC on lateral radiographs (Spearman’s rho = -0.54), and a moderate positive correlation between peak negative displacement in the PLM segment and IBC (Spearman’s rho = 0.54) in type 1 curves, all correlations between the twelve segmental peak positive and negative displacements and IBC on lateral images were weak or negligiblefor both type 1 and 5 curves (Spearman’s rho < 0.50).

## Discussion

The purpose of this pilot study with CAD/CAM technology was to provide a first impression on the effect of increased or decreased torso asymmetry and segmental peak positive or negative torso displacements on radiographic IBC in patients with AIS. The results of this study suggest that the degree of torso asymmetry correlates weakly with pre-brace major curve Cobb angle on a coronal view for both Lenke type 1 and 5 curves, and does little or negligibly correlate with IBC. Regarding the segmental peak torso displacements, only the peak negative torso displacement in the ARM segment had a strong negative correlation with IBC in type 1 curves (Spearman’s rho = -0.72) and the peak negative torso displacement in the PLM segment had a strong negative correlation with IBC (Spearman’s rho = -0.85) in type 5 curves. These results indicate that a larger expansion zone in the ARM segment is associated with less IBC in thoracic right-convex Lenke type 1 curves, and that a larger expansion zone in the PLM segment is associated with less IBC in lumbar left-convex Lenke type 5 curves.

In literature, lumbar flexion, transverse forces applied by foam pads according to the 3 or 4 pressure point principle, and total contact fit of the brace are described as mechanisms to achieve curve correction [2, 6].Using this pressure point principle, one would expect that curve correction in type 1 curves are associated to peak positive displacements in the posterior right upper segment (PRU) and anterior left upper segment (ALU), and in type 5 curves to peak positive displacements in the PLM and ARM segments. However, only weak (PRU, ARM) or weak negative correlation (ALU, PLM) with IBC were observed for these segments. On the other hand, the observed strong negative correlation between the peak negative torso displacement in the PLM segment and IBC in lumbar left-convex Lenke type 5 curves (Spearman’s rho = -0.85)could be explained by the expectation that the PLM segment should be a “pressure zone” and not an “expansion zone” according this pressure point principle. For the peak negative displacements (expansion zone), it was hypothesized that IBC was associated with peak negative displacements in the PLU and ARU for type 1 curves, and in the anterior left midsegment (ALM) and PRM segments for the type 5 curves. Also for these segments only weak (ALM) or negligible (PLU, ARU, PRM) correlation were seen with IBC. A possible explanation for the weak and negligible correlations is that peak positive displacement does not correlate with amount of applied *pressure.* A comparable amount of displacement directly applied on bones, for instance, would result in a larger spinal torso displacement compared to the same displacement on fat tissue. So far, there is insufficient evidence in literature that the magnitude of the corrective force over brace pads is correlated to the degree of radiographic IBC [20–23]. To obtain a better understanding of the correction mechanisms of the brace, future studies should focus on combined analysis of the peak positive displacement of the brace and pressure forces applied to the torso.

### Clinical implications

Identifying parameters obtained from 3D surface scans which influence IBC would be very useful in daily practice in order to investigate the effect of brace modifications on IBC and subsequently long-term brace treatment success. Based on the results of this pilot study, the degree of torso asymmetry and segmental peak torso displacements in the brace alone are not helpful in predicting IBC. It is, however, possible that when segmental peak torso displacements in-brace are combined with other factors such as pad pressure, they could be of added value in predicting IBC and/or improving brace comfort. Future studies on CAD brace related factors that influences IBC should therefore include both quantifiable parameters obtained from 3D surface scans and brace models, and pad pressure parameters in-brace obtained with electronic pressure sensors [20, 23]. In these future studies, bending radiographs before brace treatment would be an interesting additional parameter to assess besides radiographic initial in-brace correction because of the strong association between curve flexibility and initial in-brace correction [5].

### Limitations

When interpreting the results of this study a few limitations should be considered. This was a pilot study with a small sample size and a potential selection bias since the first 25 patients who gave their informed consent were included in this study. The mean initial in-brace correction of the studied group was relatively small compared to literature [24]. Once fabricated, these in-brace correction were further improved by applications of pressure pads in the brace. Therefore, these corrections only represent the CAD part of the correction. For this study it was, however, more interesting to observe the direct results of the braces fabricated with CAD technology and not with manual adjustments by the orthotist. The absence of manual adjustments by the orthotist could therefore be the reason for this relatively small in-brace correction. A limitation of dividing the 3D surface scan in twelve equally divided parts is that peak pressure points of the brace on curve apices and therefore possibly also peak displacement points might fall in different segments as a result of the variety of curve deformities. But on the other hand, dividing the 3D surface scan in anatomical sections would bring diversity in segment sizes, would be labour-intensive and possibly affect reproducibility since it must be performed manually.

In conclusion, this pilot study shows that the degree of torso asymmetry in AIS patients with Lenke type 1 and 5 curves is weakly correlated with patient’s pre-brace major curve Cobb angle on a coronal radiograph and negligible correlated with major curve IBC. Besides a strong negative correlation between peak negative torso displacement in the ARM segment and IBC in thoracic right-convex Lenke type 1 curves, and a strong negative correlation between the peak negative torso displacement in the PLM segment and IBC in lumbar left-convex type 5 curves, only some moderate, and mostly weak or negligible correlations were observed between IBC and the other segmental peak displacements for both Lenke type 1 and 5 curves. A possible explanation for the strong negative correlation between peak negative torso displacement in the PLM segment and IBC in type 5 curves is the expectation that the PLM segment should be a “pressure zone” and not an “expansion zone” according the pressure point principle.

The general results of this study indicate that the degree of torso asymmetry and segmental peak torso displacements in the brace model alone are not clearly associated with IBC. Therefore, it is highly probable that other brace related factors such as pad pressure parameters contribute to better prediction and further improvement of IBC.

## Supplementary Information

**Additional file 1: Supplementary data 1.** Correlations between segmental peak positive and negative torso displacements and initial in-brace correction on lateral radiographs in Lenke 1 curves. **Supplementary data 2. **Correlations between segmental peak positive and negative torso displacements and initial in-brace correction on lateral radiographs in Lenke 5 curves.

## Data Availability

The datasets used and/or analysed during the current study are available from the corresponding author on reasonable request.

## Abbreviations

AIS: Adolescent idiopathic scoliosis
IBC: Initial in-brace correction
3D: Three dimensional
AP: Anteroposterior
SD: Standard deviation
CAD/CAM: Computer-aided design and manufacturing systems
FEM: Finite element models
ICP: In-software iterative closest point
ARM: Anterior right midsegment
PLM: Posterior left midsegment
ALU: Anterior left upper segment
ARU: Anterior right upper segment
ALM: Anterior left midsegment
ALL: Anterior left lower segment
ARL: Anterior right lower segment
PLU: Posterior left upper segment
PRU: Posterior right upper segment
PRM: Posterior right midsegment
PLL: Posterior left lower segment
PRL: Posterior right lower segment

## References

[1] Weinstein SL, Dolan LA, Wright JG (2013). Effects of bracing in adolescents with idiopathic scoliosis. N Engl J Med.

[2] Labelle H, Bellefleur C, Joncas J et al (2007) Preliminary evaluation of a computer-assisted tool for the design and adjustment of braces in idiopathic scoliosis: a prospective and randomized study. Spine (Phila Pa 1976) 32:835–843. 10.1097/01.brs.0000259811.58372.8700007632–200704150–00002 10.1097/01.brs.0000259811.58372.87 17426626

[3] Hall JE, Miller ME, Shumann W, Stanish W (1975). A refined concept in the orthotic management of scoliosis. Orthop Prosthet.

[4] van den Bogaart M, van Royen BJ, Haanstra TM (2019). Predictive factors for brace treatment outcome in adolescent idiopathic scoliosis: a best-evidence synthesis. Eur Spine J.

[5] Peeters CMM, Hasselt AJ, Wapstra FH (2021). Predictive factors on initial in-brace correction in idiopathic scoliosis: a systematic review. Spine.

[6] Aubin CE, Dansereau J, de Guise JA et al (1997) Rib cage-spine coupling patterns involved in brace treatment of adolescent idiopathic scoliosis. Spine (Phila Pa 1976) 22:629–635. 10.1097/00007632-199703150-00010. 10.1097/00007632-199703150-00010 9089935

[7] Willner S (1984). Effect of the Boston thoracic brace on the frontal and sagittal curves of the spine. Acta Orthop Scand.

[8] Wong MS, Cheng JC, Lo KH (2005). A comparison of treatment effectiveness between the CAD/CAM method and the manual method for managing adolescent idiopathic scoliosis. Prosthet Orthot Int.

[9] Sankar WN, Albrektson J, Lerman L (2007). Scoliosis in-brace curve correction and patient preference of CAD/CAM versus plaster molded TLSOs. J Child Orthop.

[10] Cobetto N, Aubin CE, Clin J (2014). Braces Optimized With Computer-Assisted Design and Simulations Are Lighter, More Comfortable, and More Efficient Than Plaster-Cast Braces for the Treatment of Adolescent Idiopathic Scoliosis. Spine Deform.

[11] Desbiens-Blais F, Clin J, Parent S (2012). New brace design combining CAD/CAM and biomechanical simulation for the treatment of adolescent idiopathic scoliosis. Clin Biomech (Bristol, Avon).

[12] Lenke LG, Betz RR, Harms J (2001). Adolescent idiopathic scoliosis: a new classification to determine extent of spinal arthrodesis. J Bone Joint Surg Am.

[13] Vidal C, Ilharreborde B, Azoulay R (2013). Reliability of cervical lordosis and global sagittal spinal balance measurements in adolescent idiopathic scoliosis. Eur Spine J.

[14] Somoskeoy S, Tunyogi-Csapo M, Bogyo C (2012). Accuracy and reliability of coronal and sagittal spinal curvature data based on patient-specific three-dimensional models created by the EOS 2D/3D imaging system. Spine J.

[15] Bartalucci C, Furferi R, Governi L (2018). A Survey of Methods for Symmetry Detection on 3D High Point Density Models in Biomedicine. Symmetry.

[16] Meijer MF, Boerboom AL, Bulstra SK (2017). Do CAS measurements correlate with EOS 3D alignment measurements in primary TKA?. Knee Surg Sports Traumatol Arthrosc.

[17] Peeters CMM, van Houten L, Kempen DHR (2021). Assessment of pedicle size in patients with scoliosis using EOS 2D imaging: a validity and reliability study. Eur Spine J.

[18] E. D. Physical therapy research In: Principles and Applications. Philadelphia: WB Saunders; 2000.

[19] Lam GC, Hill DL, Le LH (2008). Vertebral rotation measurement: a summary and comparison of common radiographic and CT methods. Scoliosis.

[20] van den Hout JA, van Rhijn LW, van den Munckhof RJ (2002). Interface corrective force measurements in Boston brace treatment. Eur Spine J.

[21] Loukos I, Zachariou C, Nicolopoulos C (2011). Analysis of the corrective forces exerted by a dynamic derotation brace (DDB). Prosthet Orthot Int.

[22] Bulthuis GJ, Veldhuizen AG, Nijenbanning G (2008). Clinical effect of continuous corrective force delivery in the non-operative treatment of idiopathic scoliosis: a prospective cohort study of the TriaC-brace. Eur Spine J.

[23] Pham VM, Houilliez A, Schill A (2008). Study of the pressures applied by a Cheneau brace for correction of adolescent idiopathic scoliosis. Prosthet Orthot Int.

[24] Negrini S, Donzelli S, Aulisa AG (2018). 2016 SOSORT guidelines: orthopaedic and rehabilitation treatment of idiopathic scoliosis during growth. Scoliosis Spinal Disord.

