# Supplementary material for: Are torso asymmetry and torso displacements in a computer brace model associated with initial in-brace correction in adolescent idiopathic scoliosis?
Source: BMC Musculoskelet Disord. 2023 May 8;24:361. doi: 10.1186/s12891-023-06440-8 (PMC10165790; doi:10.1186/s12891-023-06440-8)
Supplement: Supplementary file 1 — Additional file 1: Supplementary data 1. Correlations between segmental peak positive and negative torso displacements and initial in-brace correction on lateral radiographs in Lenke 1 curves. Supplementary data 2. Correlations between segmental peak positive and negative torso displacements and initial in-brace correction on lateral radiographs in Lenke 5 curves. [file 12891_2023_6440_MOESM1_ESM.docx]

**Supplementary data 1:** Correlations between segmental peak positive and negative torso displacements and
 initial in-brace correctionon lateral radiographs in Lenke 1 curves.

| **Correlation with IBC** | **Little if any correlation rho≤0.25** | **Weak  correlation rho=0.26-0.49** | **Moderate correlation rho=0.50-0.69** | **Strong correlation rho= 0.70-0.89** |
| --- | --- | --- | --- | --- |
| **Peak positive torso displacement** |  |  |  |  |
| ALU | -0.18 |  |  |  |
| ARU | -0.04 |  |  |  |
| ALM |  | -0.27 |  |  |
| ARM | -0.08 |  |  |  |
| ALL |  |  | -0.54 |  |
| ARL |  | -0.48 |  |  |
| PLU | -0.24 |  |  |  |
| PRU | 0.04 |  |  |  |
| PLM | 0.24 |  |  |  |
| PRM |  | -0.36 |  |  |
| PLL | 0.16 |  |  |  |
| PRL | -0.03 |  |  |  |
| **Peak negative torso displacement** |  |  |  |  |
| ALU | 0.02 |  |  |  |
| ARU | 0.04 |  |  |  |
| ALM | 0.22 |  |  |  |
| ARM | 0.25 |  |  |  |
| ALL | -0.16 |  |  |  |
| ARL | 0.12 |  |  |  |
| PLU |  | 0.31 |  |  |
| PRU |  | 0.47 |  |  |
| PLM |  |  | 0.54 |  |
| PRM |  | 0.27 |  |  |
| PLL |  | 0.39 |  |  |
| PRL |  | 0.31 |  |  |

*Abbreviations: IBC, initial in-brace correction; rho, Spearman’s rho; ALU, anterior left upper segment, ARU, anterior right upper segment; ALM, anterior left midsegment; ARM, anterior right midsegment; ALL, anterior left lower segment; ARL, anterior right lower segment; PLU, posterior left upper segment, PRU, posterior right upper segment; PLM, posterior left midsegment; PRM, posterior right midsegment; PLL, posterior left lower segment; PRL, posterior right lower segment.*

**Supplementary data 2:** Correlations between segmental peak positive and negative torso displacements and
 initial in-brace correctionon lateral radiographs in Lenke 5 curves.

| **Correlation with IBC** | **Little if any correlation rho≤0.25** | **Weak  correlation rho=0.26-0.49** | **Moderate correlation rho=0.50-0.69** | **Strong correlation rho= 0.70-0.89** |
| --- | --- | --- | --- | --- |
| **Peak positive torso displacement** |  |  |  |  |
| ALU | -0.21 |  |  |  |
| ARU | 0.11 |  |  |  |
| ALM | -0.19 |  |  |  |
| ARM |  | -0.32 |  |  |
| ALL | -0.15 |  |  |  |
| ARL | -0.06 |  |  |  |
| PLU | 0.10 |  |  |  |
| PRU | -0.20 |  |  |  |
| PLM | -0.20 |  |  |  |
| PRM | 0.06 |  |  |  |
| PLL |  | 0.33 |  |  |
| PRL | 0.16 |  |  |  |
| **Peak negative torso displacement** |  |  |  |  |
| ALU | 0.02 |  |  |  |
| ARU | 0.06 |  |  |  |
| ALM | -0.13 |  |  |  |
| ARM | 0.09 |  |  |  |
| ALL | -0.12 |  |  |  |
| ARL | 0.03 |  |  |  |
| PLU | -0.20 |  |  |  |
| PRU |  | -0.42 |  |  |
| PLM | 0.15 |  |  |  |
| PRM | 0.22 |  |  |  |
| PLL | -0.09 |  |  |  |
| PRL | -0.20 |  |  |  |

*Abbreviations: IBC, initial in-brace correction; rho, Spearman’s rho; ALU, anterior left upper segment, ARU, anterior right upper segment; ALM, anterior left midsegment; ARM, anterior right midsegment; ALL, anterior left lower segment; ARL, anterior right lower segment; PLU, posterior left upper segment, PRU, posterior right upper segment; PLM, posterior left midsegment; PRM, posterior right midsegment; PLL, posterior left lower segment; PRL, posterior right lower segment.*
